# OER activity manipulated by IrO_6_ coordination geometry: an insight from pyrochlore iridates

**DOI:** 10.1038/srep38429

**Published:** 2016-12-02

**Authors:** Wei Sun, Ji-Yuan Liu, Xue-Qing Gong, Waqas-Qamar Zaman, Li-Mei Cao, Ji Yang

**Affiliations:** 1 State Environmental Protection Key Laboratory of Environmental Risk Assessment and Control on Chemical Processes, School of Resources and Environmental Engineering, East China University of Science and Technology, 130 Meilong Road, Shanghai 200237, P. R. China; 2 Key Laboratory for Advanced Materials, Center for Computational Chemistry and Research Institute of Industrial Catalysis, East China University of Science and Technology, 130 Meilong Road, Shanghai 200237, P. R. China

## Abstract

The anodic reaction of oxygen evolution reaction (OER), an important point for electrolysis, however, remains the obstacle due to its complicated reaction at electrochemical interfaces. Iridium oxide (IrO_2_) is the only currently known 5d transition metal oxide possessing admirable OER activity. Tremendous efforts have been carried out to enhance the activity of iridium oxides. Unfortunately there lies a gap in understanding what factors responsible for the activity in doped IrO_2_ or the novel crystal structure. Based on two metallic pyrochlores (Bi_2_Ir_2_O_7_ and Pb_2_Ir_2_O_6.5_) and IrO_2_. It has been found that there exists a strong correlation between the specific OER activity and IrO_6_ coordination geometry. The more distortion in IrO_6_ geometry ascends the activity of Ir sites, and generates activity order of Pb-Ir > IrO_2_ > Bi-Ir. Our characterizations reveal that distorted IrO_6_ in Pb-Ir induces a disappearance of J = 1/2 subbands in valence band, while Bi-Ir and IrO_2_ resist this nature probe. The performed DFT calculations indicated the distortion in IrO_6_ geometry can optimize binding strength between Ir-5d and O-2p due to broader d band width. Based on this insight, enhancement in OER activity is obtained by effects that change IrO_6_ octahedral geometry through doping or utilizing structural manipulation with nature of distorted octahedral coordination.

Over the past few years, the growing demands of decreasing greenhouse emissions and ever-increasing environmental problems drive extensively through the phase of renewable energy production and storage technologies^1^,^2^. Hydrogen (H_2_), a clean energy has been attracted much attention^3^, that can be collected by electrolytic splitting of water. This is an efficient pathway, however, is restricted intensively by the oxygen evolution reaction (OER) on the anodic surface due to its sluggish kinetics and complicated reaction mechanism^4^,^5^,^6^,^7^. A critical requirement for overcoming this bottleneck is in pursuit of developing efficient OER catalysts for decreasing the prohibitive over-potential within the quest of current density. There lies no doubt in relating the OER catalytic activity of materials to their electronic structure as bond making or breaking in OER processes are based upon the intermediates bonding of O-2p with the surface sites of catalysts^8^,^9^. Accordingly, there lie tremendous endeavors to design and prepare an efficient catalyst from both experimental and computational insights. Suntivich^10^ *et al*. descripts a strong interplay between the e_g_ antibonding occupation and OER activity tendency in perovskites, and points out e_g_~1 comprising optimum occupation. In particular, the property-activity relationship for metal oxides OER is the typical Volcano descriptor which elucidates that adsorption of O-intermediates on surface sites should be neither too strong nor too weak^11^,^12^,^13^. The density functional calculation (DFT) gives a very instructive scaling relation that the energy gap between the G_OOH*_ (the third step of OER in acid solution) and G_OH*_ (the first step of OER) is approximately about 3 eV^14^,^15^, and combines a lot of experimental results with the theoretical calculations to put forward that energy gap relative to ideal value (2.46 eV) depends on the nature of surface sites.

Currently iridium oxide (IrO_2_) is the only known 5d transition metal oxide exhibiting an admirable OER activity and being widely accepted as state-of-the-art catalyst^11^,^16^,^17^,^18^. The preciousness and expensiveness of Ir limit its expansion in various applications. Thus, a vast research is being conducted in devotion to explore an advancement in OER activity to reduce Ir consumption based on iridate, such as binary or ternary oxides by doping other metal elements like Co^19^, Ni^20^, Cu^21^, Ru^22^ and Sn^23^, or oxides of new structure like hollandite^24^ and pyrochlore^25^. Even though enhancement of OER activity is acquired through addition of elements, still there is less understanding of structural aspects demonstrating variation in its activity when transition metals replace Ir sites in rutile IrO_2_ or in the other novel crystal structure. At present, due to the unique electronic properties of Ir, the 5d iridium oxides (Iridates) are grasping more attention from magnetism and conductivity except in electro catalysis applications. For example, the perovskites iridates Sr_2_IrO_4_^26^,^27^,^28^ and CaIrO_3_^29^ are a Mott insulator due to a strong spin-orbital interaction (SOI) coupled with electron-electron repulsion in their IrO_6_ coordination to generate a band gap. While, most of iridates are metallic oxides and on account of extensive broad *d* band structure makes Fermi level (E_F_) easily crosses their valence band^30^. Therefore, there is no doubt that the different properties of iridates are completely dominated by their electronic structure, which duly has strong relationship with the IrO_6_ coordination geometry in the oxides. However, there exists a gap in understanding the correlation between IrO_6_ structure and its OER activity.

Pyrochlore metal oxides with the general formula A_2_B_2_O_7-δ_ are very common in chemistry oxides as the perovskites are the focus of attention. Their unique properties such as magnetic behavior, conductivity^31^ and phase tolerance^32^ renderenormous applications in magnetic devices^33^, Li-O_2_ battery^34^, solid state high temperature fuel cells (SOFC)^35^, nuclear waste immobilization^36^ and water splitting^32^ and so on. There are many pyrochlore iridates (A_2_Ir_2_O_7-δ_), but A when lanthanides (Nd-Yb) show a metal insulator or a metal insulator transition as a function of temperature^33^, thus they are out of our considerationas the electrical conductivity of a material is the fundamental factorfor an electrocatalyst. In here, based on two different pyrochlore iridates (Pb_2_Ir_2_O_6.5_ and Bi_2_Ir_2_O_7_), it is illustrated that the properties of IrO_6_ coordination geometry in oxides play an important role in its OER activity.

## Results and Discussion

### Physical characterization for Pb-Ir pyrocholre

**Figure 1:**
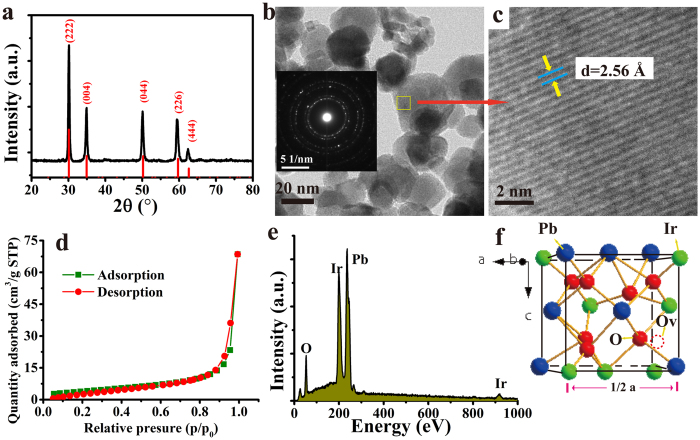
(**a**) XRD pattern of prepared Pb-Ir pyrochlore. The vertical red line corresponding to reflections of cubic structure (space group *Fd3m*) pyrochlore Pb_2_Ir_2_O_6.5_. (**b**) TEM image of Pb-Ir powder, the insert is the SAED image. (**c**) HRTEM image of Pb-Ir, the lattice fringes of (222) is indicated. (**d**) Performed isotherm linear plotof Pb-Ir. (**e**) EDS spectrum of Pb-Ir, the main elements are indicated. (**f**) 1/8 unit cell structure of Pb-Ir pyrochlore, the 8a oxygen vacancy site is marked.

The Fig. 1a shows the XRD pattern of the obtained Pb-Ir pyrochlore. All reflections of Pb-Ir indicate the presence of peak angles corresponding to the cubic pyrochlore structure (A_2_B_2_O_6_O’). The cubic pyrocholre belongs to space group Fd3m (No. 227)^37^. Generally, the larger cation A often occupies 16d site (0.5, 0.5, 0.5) and smaller cation B at 16c (0, 0, 0). The O anion resides at 48 f (x, 0.125, 0.125) and O’ anion at 8b site (0.375, 0.375, 0.375). For Pb-Ir pyrochlore, half of O’ sites are vacant (8d sites), which are marked out in Fig. 1f. Actually, the pyrochlore structure is a superstructure derivative from the fluorite^38^, the diffractions of plane angles are very close to the fluorites. It will be discussed in below part. The morphology of Pb-Ir pyrocholre is presented by TEM image shown in Fig. 1b, they are irregular nanoparticles and size is in range of 10–40 nm. The insert is the selected area electron diffraction (SAED) displaying a diffraction ring specifically a polycrystalline structure of the material. The high resolution TEM (HRTEM) reveal that the finger space is 2.57 corresponding to the (004) plane. The Fig. 1d is the linear plot of performed nitrogen adsorption isotherm BET which reveals the surface area of 14.8 m^2^ g^−1^. The surface area of IrO_2_ and Pb-Ir in different compositions are also been determined, but no correlation has been observed between composition and BET surface. The EDS curve reveals that the elemental mole ratio in Pb-Ir pyrochlore is close to stoichiometric ratio of 1:1 for Pb/Ir.

In order to have a better understanding the role of Pb in Pb-Ir oxides (include Pb-Ir pyrochlore), the different mole ratios of Pb/Ir with 1/9 (named as Pb-1), 2/8 (as Pb-2), 3/7 (as Pb-3), 5/5 (Pb-Ir pyrochlore as Pb-4), 7/3 (as Pb-5) and 9/1 (as Pb-6) are prepared, respectively. There is a plausible probability for forming solid solution oxides between Pb and Ir, because oxides of both metals have a rutile structure. However, according to the Hume-Rothery rule, the difference of ionic radius between Pb and Ir (in an octahedral) is 19.6% which is larger than15% indicating rare possibility of forming Pb-Ir rutile structure solid solution. In our study, a mixture phase comprising IrO_2_ and Pb-Ir pyrochlore is observed in Pb-2 and Pb-3 cases where Ir is rich in oxides; it ascertains that Pb cannot substitute the Ir lattice to form the rutile structure. While, an interesting feature is that Ir can be doped into PbO_2_ fluorite structure. These observations are displayed in TEM images and XRD pattern as shown in Figure S1. The morphology of IrO_2_ nanoparticle looks like a rice grain, and the nanoparticle size is about 5–10 nm, while Pb-Ir pyrochlore particle size is larger than IrO_2_, mainly due to Pb-Ir pyrochlore cell unit volume (a = 10.26 Å, V = 1081 Å^3^) being quite greater than IrO_2_ (a = 4.5 Å, c = 3.15 Å, V = 64.1 Å^3^). Thus, nanoparticles of IrO_2_ and Pb-Ir pyrochlore can be observed clearly in the TEM images of Pb-2 and Pb-3 cases. These observations are also confirmed by XRD. The peaks intensity of diffraction planes (222) and (044) for Pb-Ir pyrochlore observed in Pb-2 case increases with Pb enrichment. In contrast to this the diffraction intensity for IrO_2_ decreases until complete disappearance in Pb-4 case. Whereas, for Pb-5 and Pb-6 cases the diffraction peaks (111), (200) and (220) define a PbO_2_ fluorite structure, and no IrO_2_ diffraction planes are observed indicating that Ir can be doped into the PbO_2_ fluorite lattice. As guided by Hume-Rothery rule, the difference of ionic radius in fluorite structure is 16% which is slightly higher than 15% limit, meaning that Ir has a large possibility to substitute Pb site. From XRD pattern, the fluorite diffraction planes in Pb-5 case such as (111) and (220) are shifted slightly to right compared to Pb-6 case. According to Bragg equation, it means that smaller Ir doped to PbO_2_ decreases the inter-planar distance.

### Electrochemical performances

To assess the electrochemical properties of as-prepared materials, the electrodes are evaluated by the typical technique of cyclic voltammetry (CV) under the 0.1 M HClO_4_ solution with different scan rates. As previously reported^39^,^40^, the voltammetric charge (q*) has a strong correlation with the active surface area. The more active sites the larger the charge. The voltammetric charge q* (mC∙cm^−2^) is obtained as follows equation 1:


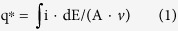


i is recored current (mA), E is the potential (V) which is a fuction of the scan rate, A is the geometry area of electrode (cm^2^) and ν is the scan rate (V s^−1^).

**Figure 2:**
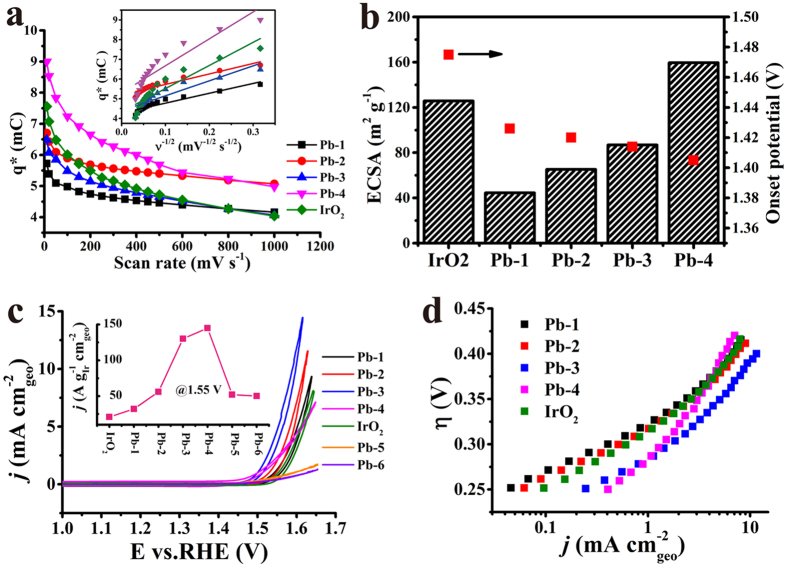
(**a**) Voltammetric charge q* at the different scan rate for Pb-1, Pb-2, Pb-3, Pb-4 and IrO_2_ as reference. The insert is q* plot as a function with ν^−1/2^. (**b**) Determined ECSA and onset potential of prepared different catalysts. Histogram corresponding to the ECSA. (**c**) Quasi-stationary OER polarization curves (*iR* corrected) of prepared Pb-Ir oxides in 0.1 M HClO_4_. The insert is their specific activity @ 1.55 V vs. RHE. The loading amount of all of the materials was 0.2 mg cm^−2^. (**d**) Determined Tafel plots of Pb-Ir oxides.

Figure 2a presents the typical trends^39^,^41^,^42^ in which q* decreases with increasing scan rates. Here, the Pb-4 obtains a higher q* than any other materials, but there is no linear relation of the q* with increasing Pb content. In detail, Pb-1 and Pb-2 have lower values than IrO_2_ does, while Pb-3 and Pb-4 samples are in contradiction. The function of q* with v^−1/2^ can give the inner capacitance of the electrodes, an important descriptor for evaluating the number of active sites by extrapolating the charge at zero sweep rate. The order of the inner capacitance is Pb-4 > Pb-2 > IrO_2_ > Pb-3 > Pb-1, which shows that Pb-Ir pyrochlore has more active sites. The electrochemical active surface area (ECSA) can be determined by using the double layer capacitance (the double layer capacitance is calculated in Figure S2). The Fig. 2b shows that Pb-4 has the largest ECSA of 160 m^2^ g^−1^ while IrO_2_ provides a medial value of 121 m^2^ g^−1^. The ECSA of Pb-Ir cases increase with Pb enrichment although being lower than IrO_2_ except Pb-4 case. It is hard to explain the ECSA variations in Pb-Ir oxides by simple physically mixed Pb-Ir pyrochlore with IrO_2_, which implies a strong interaction between the oxides in low Pb contents.

In order to estimate their OER activity, firstly, their onset potential by normalizing the current (see detail in SI Figure S2) determined by CV methods are determined. As shown in Fig. 2b, the onset potential of Pb-4 is very low executing only 1.405 V that is quite less than IrO_2_ at 1.475 V. In further, the onset potential of Pb-Ir oxides presents a decreasing trend on increasing Pb contents, signifying Pb-Ir pyrocholre as a water oxidation catalyst. The polarization curves of OER are presented in Fig. 2c, it can be found that Pb-3 has a notable OER activity. In low Pb composite cases, the OER activity enhances with increasing Pb content. The Pb-4 is the watershed, in low Ir composite cases, such as Pb-5 and Pb-6 show low OER response that indicates Ir as a real active site rather than Pb. However, there is no doubt that strong interaction of the two phases has a remarkable synergistic effect to enforce the OER activity. The Tafel plots (Fig. 2d) confirm the observations, where Pb-3 gives 59 mV dec^−1^ while the benchmark IrO_2_ presents 72 mV dec^−1^. In a pity, the Pb-4 only affords 93 mV dec^−1^. However, as discussed above, Ir is the only active site, their mass specific activity by using the mass of Ir at the 1.55 V (η = 0.32 V, a mediate over potential). In the insert of Fig. 2c, the Pb-4 displays the highest specific activity, which further confirms that Pb-Ir pyrochlore is a good candidate for OER.

Another noted metallic Ir pyrochlore structure, Bi_2_Ir_2_O_7_ (Bi-Ir) oxide also been prepared via hydrothermal synthesis. The XRD (as shown in Figure S3) confirms it has a cubic pyrochlore structure similar to Pb-Ir composite. The outstanding OER performance of Pb-Ir pyrochlore out reaches Bi-Ir composite. As shown in Figure S4, Bi-Ir exhibits very poor OER activity, and its specific activity remains lowest as well. This finding is analogous to Javier Parrondo *et al*.^43^ results, while Kripasindhu Sardar *et al*.^44^ found the Bi-Ir can afford a good OER activity. The contradiction may mainly due to the different catalyst loading and employed electrolyte solution (for Sardar’s results tested under 1 M H_2_SO_4_ instead of 0.1 M HClO_4_).

### XPS and VBS characterizations

**Figure 3:**
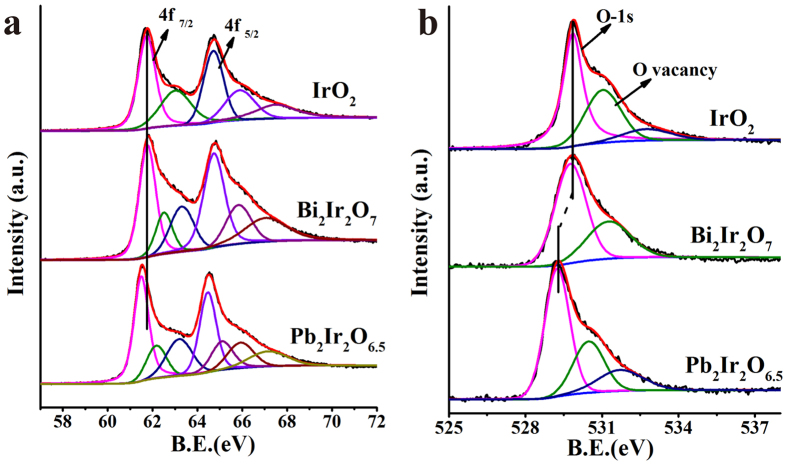
(**a**) Ir-4f and (**b**) O-1s core level XPS spectra of IrO_2_, Bi-Ir and Pb-Ir pyrochlores. The binding energy shift is indicated by vertical line.

In here, the extremely different OER performances of these three oxides (IrO_2_, Pb-Ir and Bi-Ir pyrochlore) are attracted our attention. No matter either computationally or experimentally, the OER activity of a material has a strong correlation with its electronic structure. Herein, it is worth to elaborate the differences in electronic structure of these oxides. Figure 3a is their Ir-4f core level X-ray photoemission spectrum (XPS). For the single crystal IrO_2_, the binding energy of Ir-4f7/2 and 4f5/2 are 61.7 eV and 64.7 eV, respectively. In our hydrothermally synthesized IrO_2_ sample, the binding energies are 61.76 and 64.7 eV, which are close to single IrO_2_ values. Comparing to IrO_2_, Pb-Ir pyrochlore shows a clear low binding energy, while there is no variation between IrO_2_ and Bi-Ir. However, our finding is different compared to Kennedys reporting partial Ir component having a higher binding energy and owing to the Ir^5+^ valence. For the extensive Ir oxides, there is distinctly asymmetrical shape of the Ir-4f photoemission spectra due to the final state effects. J. M. Kahk *et al*.^30^ systematically studied this asymmetric shape in IrO_2_ and proposed that it was incorrectly attributed to the complex line of different valence states, especially for those containing higher valence surface phase. Seemingly, in our de-convoluted Ir-4f spectra for IrO_2_ case, there exists a higher binding energy peak. As precisely because of the strong asymmetric peak shape exhibited in Ir-4f, it is no longer tenable in regarding the higher binding energy as the higher valence state. In similarity, the de-convoluted peaks with higher binding energy in Bi-Ir and Pb-Ir are not contributed to the high Ir valence phase.

The O-1s core level XPS displayed in Fig. 3b are also been compared. It clearly depicts a marked shift towards lower binding energy in Pb-Ir compared to IrO_2_. While for Bi-Ir case, the O-1s has no significant change compare to that of IrO_2_. The binding energy of Bi-4f_7/2_ is 158.05 eV (as shown in Figure S5), which is close to the value reported by Kripasindhu Sardar^44^ *et al*., suggesting the presence of Bi with +3 valence in here. The binding energy of three elements in all prepared Pb-Ir oxides are compared as shown in Figure S6. From Pb-4f XPS, it is clearly noted that two de-convoluted peaks were identified in Pb-Ir oxides except Pb-1. These doublet peaks are also observed in β-PbO_2_, but their peak shapes are different^45^. For in β-PbO_2_, it is a tetragonal structure. While in our samples, Pb is located in cubic pyrochlore structure. Their different crystal structure is responsible for their novel peak distribution. It is noted that the binding energy of Pb-4f progressively shifts to a higher energy direction with increasing Pb component until Pb-4 case. When Pb content further increases such as in Pb-5 and Pb-6, the binding energy shifts to a lower value. The O-1s are also complicated. In Pb-2 case, three fitting peaks are observed, the low energy one may contribute to Pb-Ir pyrochlore, while the middle one arises from binding of Ir atom which is consistent with IrO_2_ reflection. To be noted, there is no significant difference in energy position of lattice O between pyrochlore and fluorite structure. For the Ir-4f XPS, there is a slight shift to lower energy in all Pb-Ir oxides even in Pb-Ir fluorite. This difference is probably due to the different crystal structure of pyrochlores and fluorites. These findings are also observed in the valence band spectra of O-2s and Pb-5d components (as shown in Figure S7), where O-2s has an inverse variation to Pb-5d whose energy position increase with increasing Pb component. However, there is no obvious variation of Ir binding energy to Pb/Ir ratio changes.

### Differences in their IrO_6_ octahedral geometry

**Figure 4:**
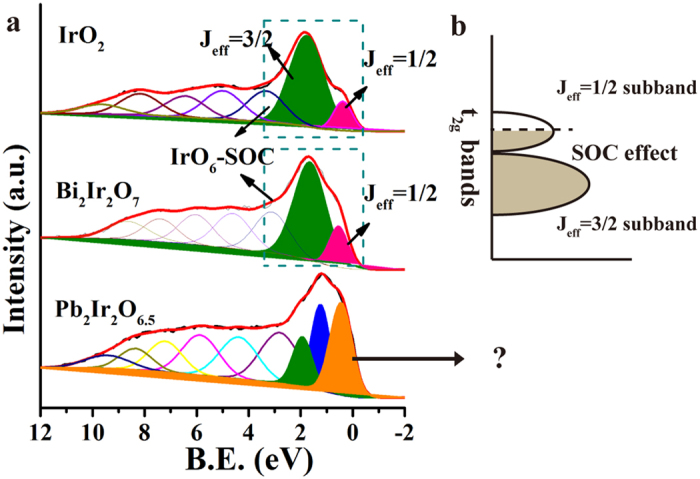
(**a**) Valence band spectra of IrO_2_, Bi-Ir and Pb-Ir pyrochlores. The t_2g_ subbands are indicated in IrO_2_ and Bi-Ir cases by fitting the peaks. (**b**) The schematic energy diagrams for Ir t_2g_^5^ configuration with SOC effect in IrO_6_ octahedral coordination.

Apart from the differences in Ir-4f XPS spectra of these three oxide types (IrO_2_, Pb-Ir and Bi-Ir), their VBS also have obvious distinction. Figure 4a is the VBS of IrO_2_, Bi-Ir and Pb-Ir by applying Shirley background. It is worth noting that a shoulder peak near Fermi level is obviously observed in IrO_2_, that attributes as spin-orbital couple (SOC) splitting the five t_2g_ bands into four electrons occupied J = 3/2 subband and one electron occupied J = 1/2 subband^27^,^29^ (note that IrO_2_ gives the electron configuration of t_2g_^5^e_g_^0^). The J = 1/2 subband formation is descripted in Fig. 4b. This shoulder peak is common in many iridium oxides and is utilized to characterize the SOC effect in IrO_6_ octahedral coordination. Many efforts proposed that a strong SOC effect and electron-electron repulsion determine the nature of Motta-insulator in Sr_2_Ir_2_O_4_ and CaIrO_3_. Despite the strong SOC (our VBS also confirms it) effect also embeds in IrO_2_, the wide t_2g_ bands and special orbital property contribute to conducting behavior of IrO_2_ (our DOS will also confirms the fact that electrons cross the Fermi level). Notable fact that this weak shoulder peak is also observed in our Bi-Ir case, the intensity of J = 1/2 subband is relatively weaker than J = 3/2 due to a quarter of electrons states^46^. However, the Pb-Ir VBS depicts a notable feature with very large shoulder peak rather than a weak shoulder peak which is in contrast to IrO_2_ and Bi-Ir. The paradox is that this J = 1/2 subband state is even higher than its neighboring J = 3/2 subband. Accordingly, it implies that the SOC effect of IrO_6_ coordination may disappear in Pb-Ir pyrochlore. The VBS for all prepared Pb-Ir oxides as shown in Figure S7. It is worth to note that the J = 1/2 subband is clearly observed in case of Pb-1, Pb-2 and Pb-3, because they are mixture of two phases of IrO_2_ and Pb-Ir pyrochlore, thus IrO_2_ contributes the shoulder peak nearly E_F_. Intriguingly, the disappearance of J = 1/2 subband is also present in Pb-5 and Pb-6 cases, it is mainly due to that the IrO_6_ symmetry of Ir replaced by IrO_8_ coordination in fluorite structure.

**Figure 5:**
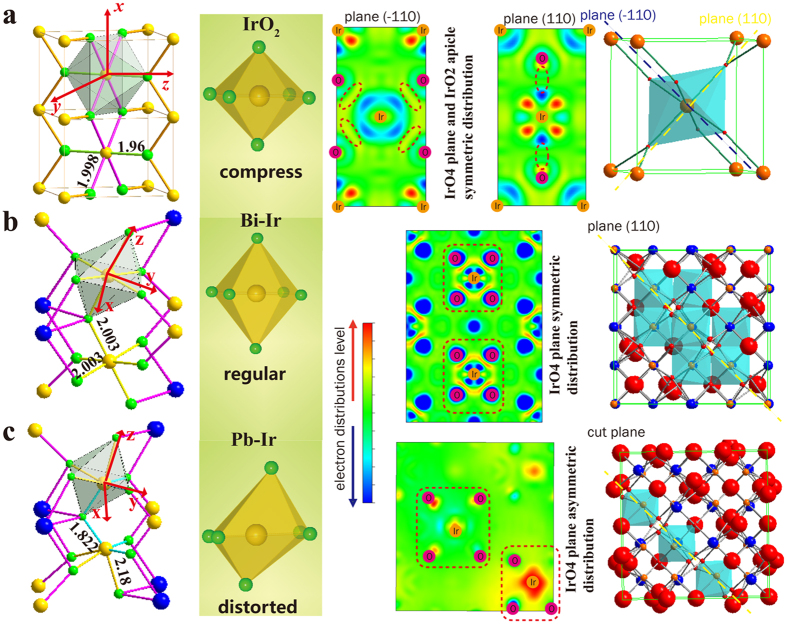
(**a**–**c**) respectively are the IrO_6_ polyhedral, the Ir-O bond length are indicated in structure, the shape of IrO_6_ octahedral coordination, lone pair charge density for the specific cut plane and the cell unit crystal structure in IrO_2_, Bi-Ir and Pb-Ir pyrochlores. The cut plane are mainly cross the four plane Ir-O bonds in Bi-Ir and Pb-Ir. The Ir and O atoms are placed in the charge density diagrams.

This extraordinary disappearance of J = 1/2 subband may arise from the degeneracy removal of the orbitals. Lifting degeneracy of orbitals in the metal-ligands is often accompanied by the structural distortion. A famous case commonly discovered on six-coordinated Cu^2+^ 47, Mn^3+^ 48 compounds is the Jahn-Teller effects, a structural distortion result of transformation from regular octahedron to elongated one, which strongly affects electronic structure in materials and consequently influence their conductivity, magnetism, optical property and catalytic activity. In here, it is noted that the IrO_6_ coordination in these three oxides is extremely different as displayed in Fig. 5. In the case of IrO_2_, the six bonds of IrO_6_ octahedron are unequal with two short apical Ir-O bond (1.96 Å) and four long planar Ir-O bond (1.998 Å), which gives a slightly compressed octahedron (transfer from O_h_ to D_2h_). While in Bi-Ir case, the six bonds are all equivalent (2.003 Å) and delineate a regular octahedron. Composites of IrO_2_ and Bi-Ir do not deviate significantly from the O_h_ symmetry and avoid large structure distortion, in consequence a strong J = 1/2 peak is observed from performed VBS. Remarkably, the octahedral coordination is intensively distorted in Pb-Ir case where the un-equivalent bonds not only exist in apical direction (one is 2.18 Å and another is 1.822 Å) but also in the four planar Ir-O bonds (two of them are 2.18 Å and the remain are 1.822 Å). Their Ir-O bond lengths distribution are also calculated based on the most stable crystal model as shown in Figure S8, the Pb-Ir composite has different Ir-O bond length both in the plane and apical section of the octahedron. This distortion will result in weakening of shielding effect in the elongated bond direction, and further lifting the t_2g_ and e_g_ degeneracy responsible for the disappearance of J = 1/2 subband. The properties associated to lone pair distribution of the later are also significantly different from IrO_2_ and Bi-Ir. It presents asymmetric and uneven distribution of charge density, whereas it is symmetric and uniform in case of IrO_2_ and Bi-Ir in their 4-coordination plane.

**Figure 6:**
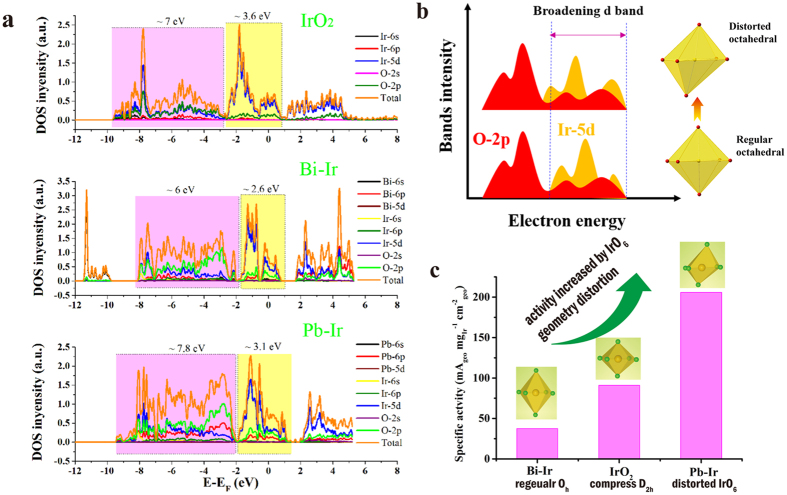
(**a**) DOS for IrO_2_, Bi-Ir and Pb-Ir pyrochlores. The colored by pink associates with bonding area, yellow one corresponding to antibonding area. (**b**) Schematic diagram of the broadening Ir-5d band induced by distorted octahedral enhancing orbitals overlap between d bands and O-2p. (**c**) The OER specific activity order of IrO_2_, Bi-Ir and Pb-Ir is in accordance with the order of IrO_6_ geometry distortion.

As demonstrated above, the nature of IrO_6_ coordination is responsible for the differences both in the VBS and charge density distribution of lone pair. The distorted IrO_6_ coordination brings a significance in bonding character and occupancy of the orbital states. Figure 6a is the calculated density of state (DOS) of the three compounds. The bonding area with pink color represents σ bonding and π bonding. It can be found clearly that Pb-Ir has the widest (7.8 eV bandwidth) bonding region, while the Bi-Ir gives the narrowest bandwidth and the IrO_2_ has the medial one. For the π anti-bonding colored by yellow, IrO_2_ provides bandwidth of 3.6 eV and Pb-Ir approaches 3.1 eV, both remaining higher than Bi-Ir. The Pb-Ir shows the broadest width in whole conduction band of 10.9 eV, which mainly is the outcome of the distorted IrO_6_ coordination. Previous studies revealed a principle^12^,^13^,^49^ for the most powerful catalyst that the M (metal) −O bond in oxides is neither too strong nor too weak so that it can tune energy for the rate-determining step (RDS). Aleksandra Vojvodic *et al*.^13^ pointed out that the occupancy of the d states correlates with the oxygen adsorption energy and hence affects the catalytic activity. The adsorption strength depends on the interaction between metal’s d bands and O-2p adsorbate. The higher degree of overlap, the stronger the bonding. As illustrated in Fig. 6b, the distorted IrO_6_ octahedron gives rise to a broaden Ir-5d bands, which improves the orbital overlap with O-2p. It further strengths the intermediate adsorbates (O*, OH* and ooh*) interacting with catalyst surface sites, especially decreasing the OOH* (often regarded as the rate-determining step) adsorption energy. In our findings, the Pb-Ir pyrochlore has the most broad bands in these three oxides, while the most regular IrO_6_ symmetry in Bi-Ir has the narrowest bands and IrO_2_ has a middle one. As shown in Fig. 6c, the OER specific activity order is Pb-Ir > IrO_2_ > Bi-Ir, which is consistent with d bands width order and also with the order of IrO_6_ geometry distortion. Thus, it is concluded that Ir-based oxides are able to enhance its OER activity by increasing IrO_6_ geometry distortion in their crystal structure.

As our previous investigation on the Cu^21^ doped IrO_2_, when the Cu replace the Ir site in IrO_2_ gives rise to a distortion of the IrO_6_ coordination due to a strong Jahn-Teller effect of CuO_6_, which further influence the electronic structure of Ir site in particular changes the distribution of 5d electrons. The hollandite structure of K_x_ = _0.25_IrO_2_^24^ also exhibits better OER activity than IrO_2_, and its IrO_6_ coordination is a distorted octahedron that strongly affect its electronic structure. Shengli Chen *et al*.^19^ reported that Co doped IrO_2_ with porous hierarchical architecture leads to a higher OER performance, their displayed XRD has an obvious shift along c axis indicating a variation of crystal lattice. P.B. Balbuena *et al*.^50^ by means of DFT calculation confirmed that Co has a significant effect in tuning the electronic structure. Tobias Reier *et al*.^20^ reported that Ir-Ni oxides give an outstanding OER activity, the O-K edge XANES also showed the changes in electrons distribution between orbitals of Ir-O bonds. All above of the transition metals Cu, Co, Ni doped into IrO_2_ brings enhancement in OER activity, mainly due to the dopant atoms altering the IrO_6_ coordination. In this circumstance of mismatched crystal systems, oxygen vacancies are induced due to the low valence dopants. As depicted from DOS of Cu doped and K_x_ = _0.25_IrO_2_ cases, their IrO_6_ octahedral coordination is also quite different from IrO_2_ which leading to a broadened valence band. Thus, these variations further lead to optimize the electronic structure resulting in promoting the OER activity of Ir sites.

In summary, we have prepared successfully Pb-Ir, Bi-Ir pyrochlores and relevant Pb-Ir oxides by simple hydrothermal synthesis and characterized their OER activity along with structural properties. The order of specific activity for OER is that Pb-Ir > IrO_2_ > Bi-Ir pyrochlore. We demonstrate that there is a strong correlation between the IrO_6_ coordination geometry in the oxides structure and their OER activity. For the Pb-Ir pyrochlore, it has a strong distorted IrO_6_ geometry due to different Ir-O bond lengths, while IrO_2_ is a D_2h_ symmetry and Bi-Ir gives a perfect IrO_6_ octahedron without any distortion. The differences in their IrO_6_ coordination also change their valence band structure causing disappearance of J = 1/2 sunbands in Pb-Ir case. The properties of IrO_6_ geometry also affect significantly their lone pair charge density distribution, where Pb-Ir has an asymmetric charge density distribution while Bi-Ir upholds symmetricity. The calculated DOS confirms that Pb-Ir has a more broaden bandwidth than IrO_2_, while Bi-Ir has the narrowest. The broaden bands no doubt promote O-2p (intermediate adsorbate) overlap with valence band of oxides, that benefits in response to enhance its OER activity. Also, previous studies on transition metals (Cu, Co, Ni) doped IrO_2_ obtain enhancements for promoting OER activity prove that there are some defects and lattice disorders due to the mismatch of the crystal structures. In short, our demonstrations indicated that the distorted IrO_6_ geometry plays an important role in determining its OER activity. Based on these results, it can be obtained that enhancement in OER activity is affected due to change in IrO_6_ octahedral geometry via doping or utilizing other structure corresponding to the nature of distorted octahedral coordination.

## Methods

### Synthesis of Pb-Ir and Bi-Ir pyrochlores

Both two pyrochlores are synthesized by using hydrothermal method. This method doesn’t need rigorous preparatory conditions and is time efficient. First, the feeding mole ratio of Pb:Ir is 1:1 by using simple precursors of Pb(NO_3_)_2_ and IrCl_3_ aqueous solution. Then the mixture is transferred into 40 mL Teflon-lined pressure vessel with adding 10 mL deionized water and 10 mL 0.5 M NaOH aqueous solution. Subsequently, the reactor is loaded into an oven heating to 250 °C for 720 min; then, the vessel is cooled naturally at room temperature. The precipitates are suction filtered and washed with deionized water twice to remove other ions. The remaining solid on the filter was dried to dehydration in an oven at 80 °C for 1 h. The dried solid was transferred to a crucible and annealed at 600 °C for 6 h to produce excellent crystallinity. For Bi-Ir case, as Bi(NO_3_)_2_ dissolutes in 1 M HNO3, thus to neutralize acid more alkali (1.2 M NaOH) was added. We also prepared different compositions of Pb-Ir oxides in order to investigate deeply the nature of Pb-Ir pyrochlore in OER.

### Characterization

The crystal structure of the catalysts were investigated using powder X-Ray diffraction (XRD) using a D/max2550 V apparatus with a Cu-Kα radiation source (λ = 1.5406 Å). The morphologies of the catalysts were observed using a field-emission scanning electron microscope (FESEM) equipped with a Nova NanoS and the Energy dispersive X-ray (EDX) spectrometer to confirm the composition using a TEAMApollo system. A JEM-2100 transmission electron microscope was used to obtain the TEM and HRTEM images. The surface properties of the catalysts were determined via X-ray photoelectron spectroscopy (XPS) using an ESCALAB 250Xi instrument. The samples were sputter coated with carbon, and the spectra were calibrated with respect to C-*1s* at a binding energy of 284.6 eV.

### Electrode Preparation and Electrochemical Measurements

In this study, the electrodes used for the electrochemical measurements are of the so-called dimensionally stable anode (DSA) type, which were prepared as follows. 6 mg of fresh catalyst powders are dispersed in 1.5 mL of 2:1 v/v isopropanol/water and then ultrasonicated for approximately 30 min to form a homogeneous ink. Next, 7.5 μL of ink deposited on 0.5 cm × 1.5 cm Ti plate, which was etched for 2 h by 10% (wt %) oxalic acid under near boiling conditions and then washed with deionized water. The process was repeated 5 times to obtain a loading weight of approximately 0.2 mg cm^−2^ and then stabilized by annealing for 20 minutes at 400 °C on each cycle. All electrochemical measurements are conducted in a three–electrode system. The working electrode used was 0.5 cm × 0.5 cm (electrode reactive area = 0.25 cm^2^) of the prepared DSA. A saturated calomel reference electrode (SCE) and a polished and cleaned Pt foil with a 1.5 cm × 1 cm reaction area were used for the counter electrode. The electrode potential from the SCE scale was converted to the reversible hydrogen electrode (RHE) scale by calibrating with:


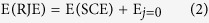


The over-potential values (η) corrected with the iR were obtained using the following equation:


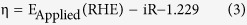


where i is the current, and R is the uncompensated Ohmic electrolyte resistance. The working electrodes were cycled several times (at least 10 times) until the curves were observed to overlap. The Tafel plots were conducted by the stair-case voltammetry method at the different potential range (vs. RHE), with 10 mV steps every 100 s (scan rate 0.1 mV/s) and current values were read at the end of each step. The electrolyte is 0.1 M HClO_4_ (pH~1).

### RHE calibration

The SCE was calibrated with respect to the RHE in all three types of pH solution using a high purity hydrogen saturated electrolyte with a Pt foil as the working electrode^51^. CVs were run at a scan rate of 1 mV/s, and the average of the two potentials at which the current crossed zero was recorded as the thermodynamic potential for the hydrogen electrode reaction.

### Computational Details

All the DFT calculations were performed by Vienna Ab-initio Simulation Package (VASP)^52^,^53^. The projector augment wave (PAW) method with the generalized gradient approximation (GGA) for the exchange-correlation functional in a form suggested by Perdew, Burke, and Ernzerho^54^ was used to optimize the lattice parameters of bulk IrO_2_, Bi_2_Ir_2_O_7_ and Pb_2_Ir_2_O_6.5_ and calculate the electronic structures. A 400 eV plane-wave cut-off energy and spin polarization was set and the residual force components on each atom are lower than 0.05 eV Å^−1^·atom^−1^. A 20 × 20 × 24 k-mesh for IrO_2_ bulk and 5 × 5 × 5 for Bi_2_Ir_2_O_7_ and Pb_2_Ir_2_O_6.5_ are employed, respectively, and the oxygen defects in Pb_2_Ir_2_O_6.5_ bulk were described as vacancies^55^. The charge density images were drawn by VESTA program^56^.

## Additional Information

**How to cite this article**: Sun, W. *et al*. OER activity manipulated by IrO_6_ coordination geometry: an insight from pyrochlore iridates. *Sci. Rep.* **6**, 38429; doi: 10.1038/srep38429 (2016).

**Publisher's note:** Springer Nature remains neutral with regard to jurisdictional claims in published maps and institutional affiliations.

## Supplementary Material

Supplementary Information
