# Supplementary material for: OER activity manipulated by IrO6 coordination geometry: an insight from pyrochlore iridates
Source: Sci Rep. 2016 Dec 2;6:38429. doi: 10.1038/srep38429 (PMC5133550; doi:10.1038/srep38429)
Supplement: Supplementary Information [file srep38429-s1.doc]

**Supplementary Information**

**OER activity manipulated by IrO6 coordination geometry: an insight from pyrochlore iridates**

**Wei Sun^1^, Ji-Yuan Liu^2^, Xue-Qing Gong^2^, Waqas-Qamar Zaman^1^, Li-Mei Cao^1^, Ji Yang***

^1^State Environmental Protection Key Laboratory of Environmental Risk Assessment and Control on Chemical Processes, School of Resources and Environmental Engineering, ^2^Key Laboratory for Advanced Materials, Center for Computational Chemistry and Research Institute of Industrial Catalysis, East China University of Science and Technology, 130 Meilong Road, Shanghai 200237, P.R. China.

*To whom correspondence should be addressed.

Corresponding authors’ E-mail: [yangji@ecust.edu.cn](mailto:yangji@ecust.edu.cn);


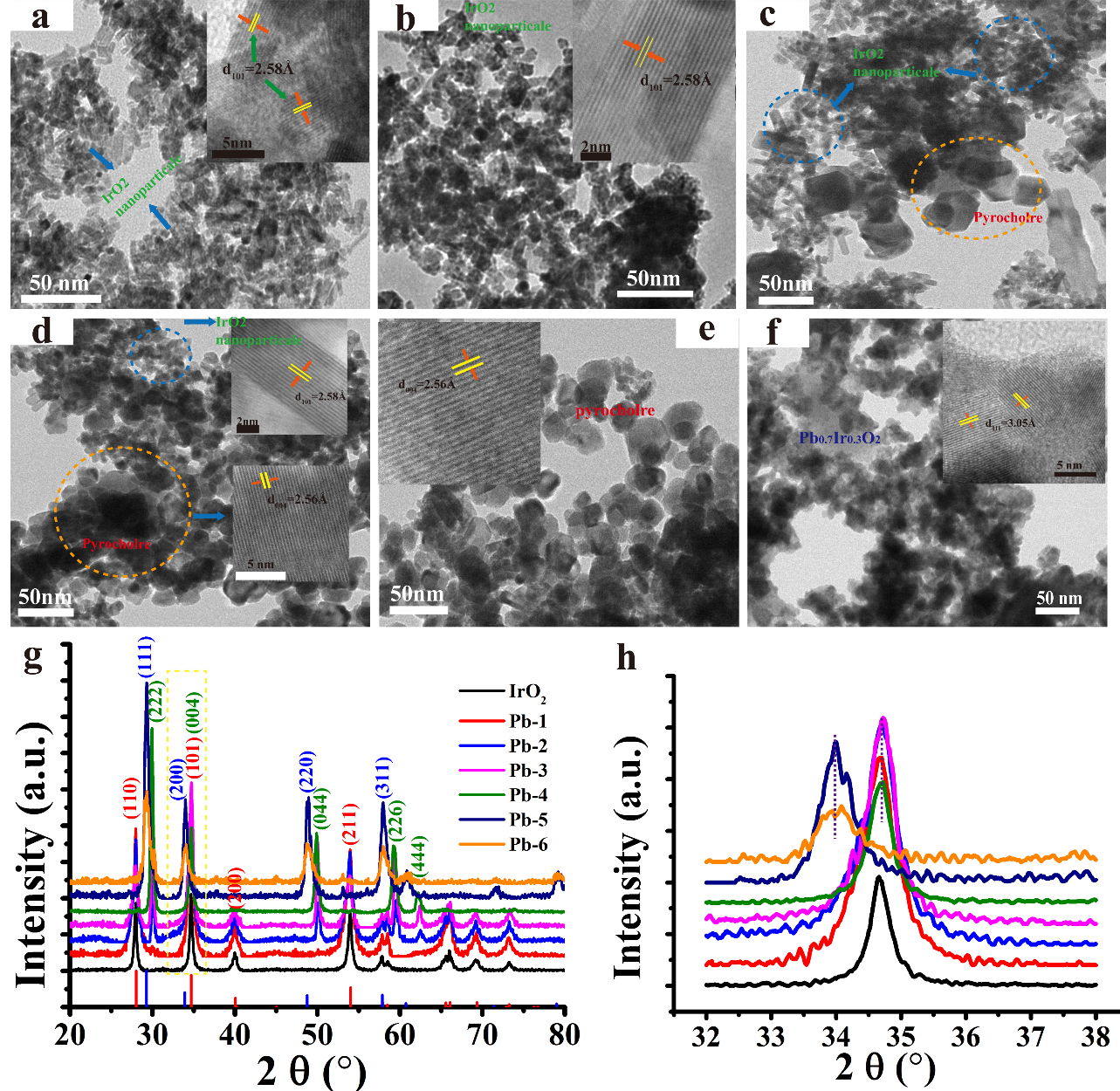


**Figure S1.** (a)-(f) are the TEM images of IrO_2_, Pb-1,Pb-2,Pb-3,Pb-4 and Pb-5, respectively. The inserts are their HRTEM and fringe spaces are indicated. It can clearly find that the nanoparticle size of IrO_2_ is smaller than Pb-Ir pyrochlore, thus it is very easy to distinguish those two oxides by TEM images. (g) and (h) are the XRD patterns of the prepared different Pb-Ir oxides. As discussed in main text, the pyrochlore derivates from the fluorite structure, thus in Pb-5 and Pb-6 cases, the (222) diffraction plane transfer to (111) plane displayed as their angle shift. The vertical red line is the IrO_2_ standard reflections, where blue one corresponds to pyrochlore structure.


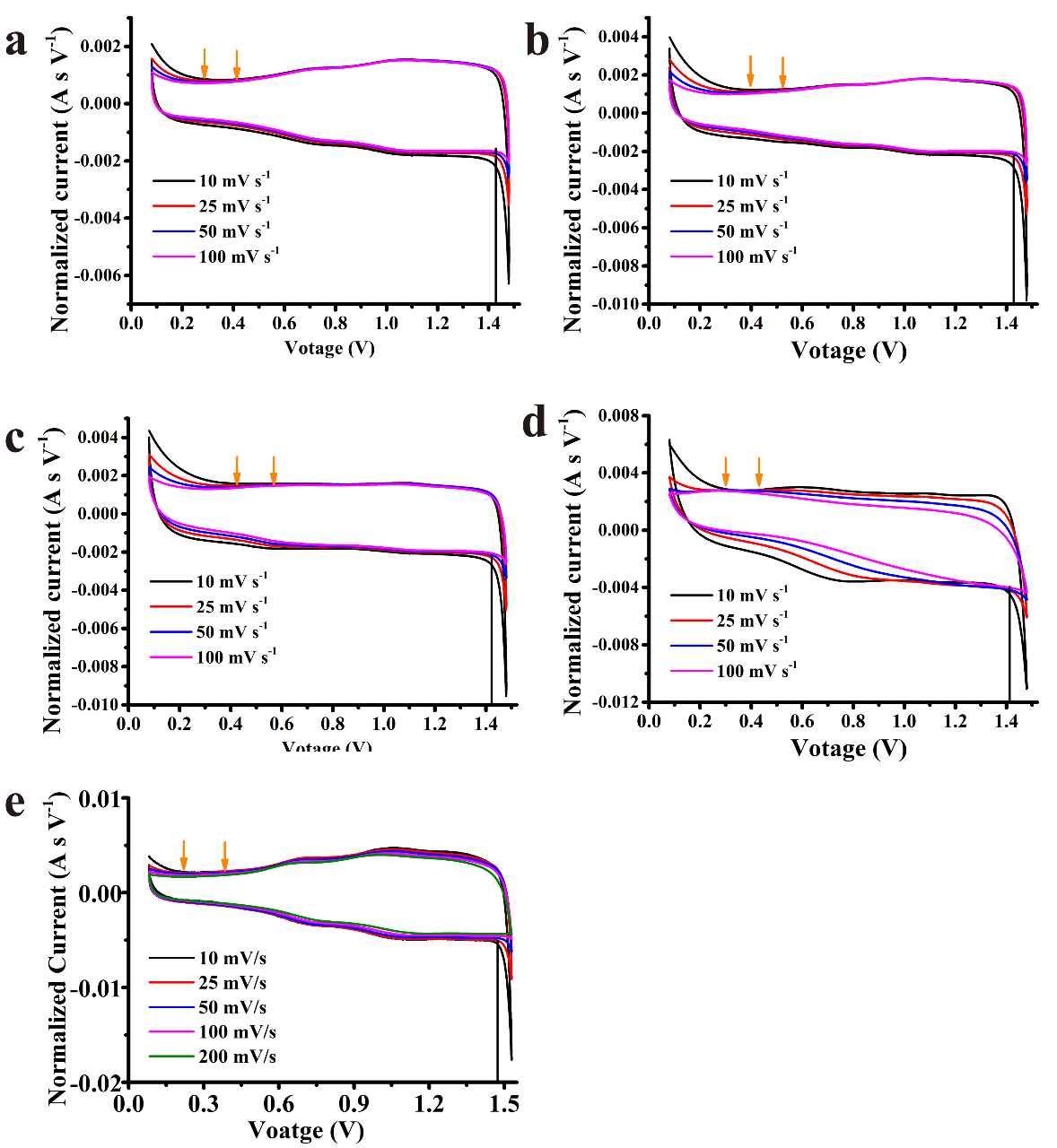


**Figure S2.** Normalized CV curves for (a) Pb-1, (b) Pb-2, (c) Pb-3, (d) Pb-4 and (e) IrO_2_. The current determined by CV method includes two parts, capacitor current (i_c_) and Faradic current (i_F_). While i_c_ is liner with scan rate by i_c_=C_d_∙ν. The parameter C_d_ is crucile factor associated with electrochemical active sites. Thus, it can be normalized by i/ν= C_d_ (constant) +i_F_/ν both obtaining the C_d_ value and marked Faradic current to determine the onset potential of OER activity. The region between vertical orange lines is chosen to estimate C_d_ value.


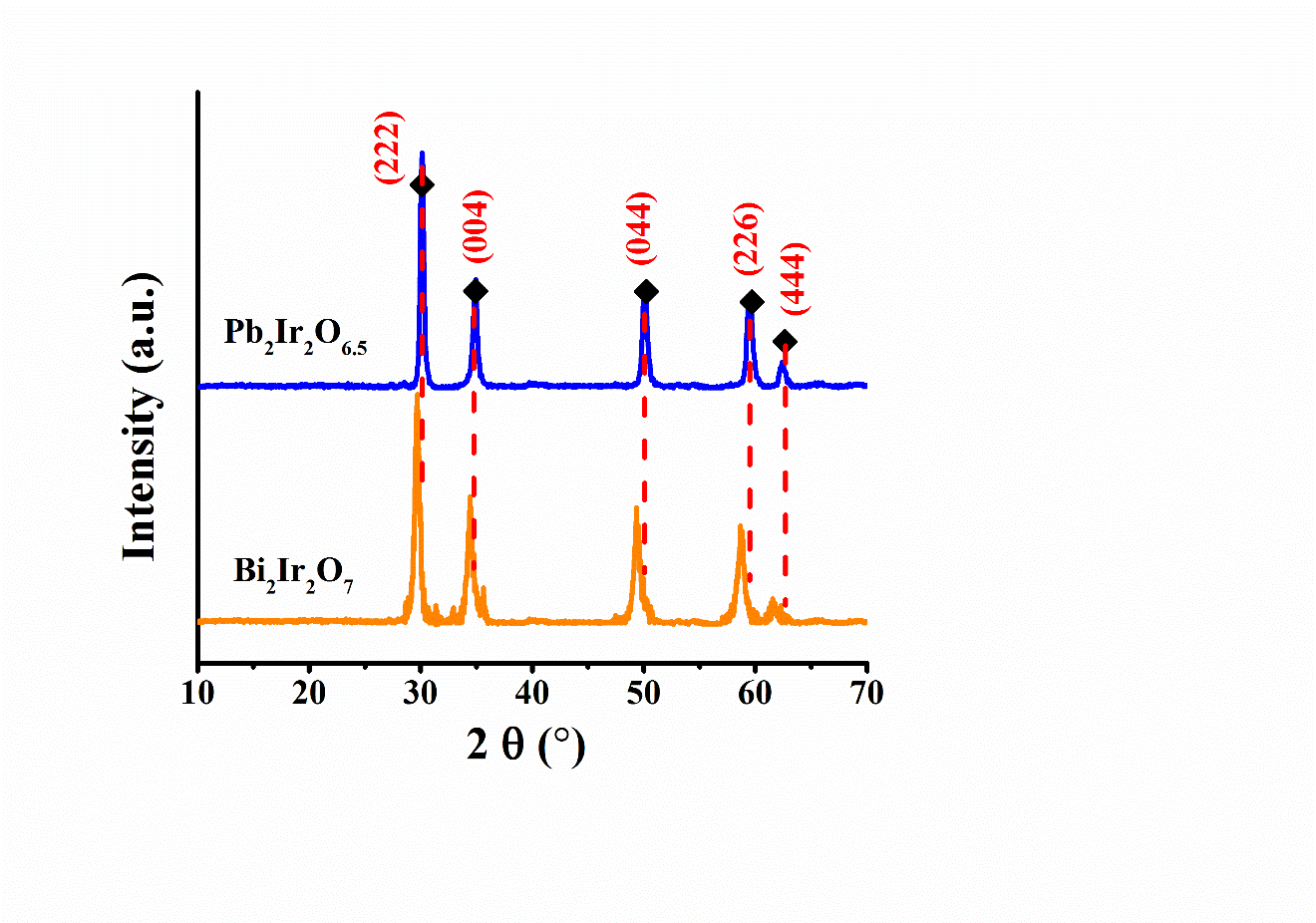


**Figure S3.** XRD patterns of Pb-Ir and Bi-Ir pyrochlores. The difference is the angle shift to smaller degree in Bi-Ir, mainly due to Bi-Ir having larger cell parameters than Pb-Ir.


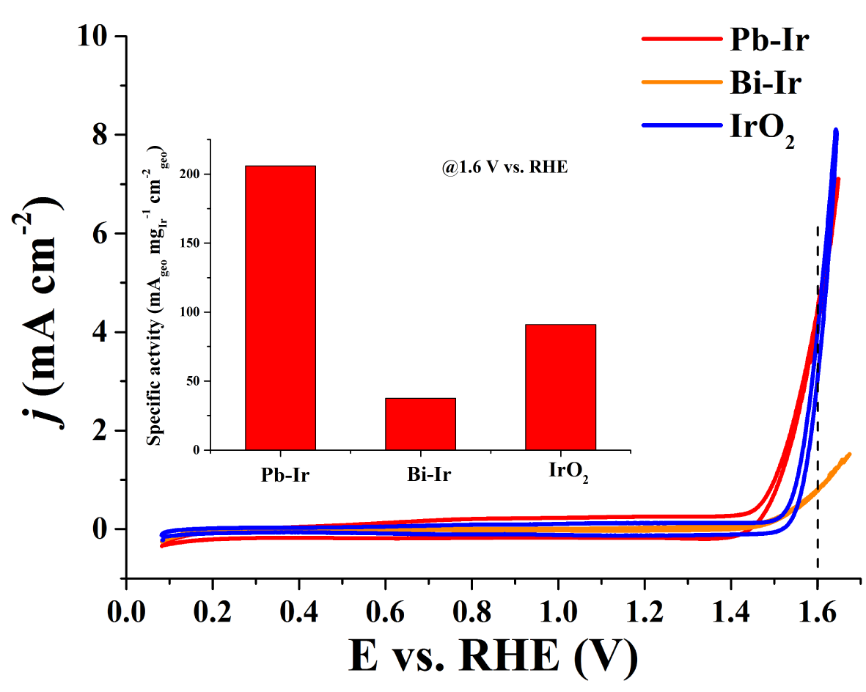


**Figure S4.** OER polarization curves of Pb-Ir, Bi-Ir and IrO_2_ with iR correction under 0.1 M HClO_4_, scan rate 10 mV s^-1^, catalyst mass loading of 0.2 mg cm^-2^. Insert is their specific activity by Ir at 1.6 V.


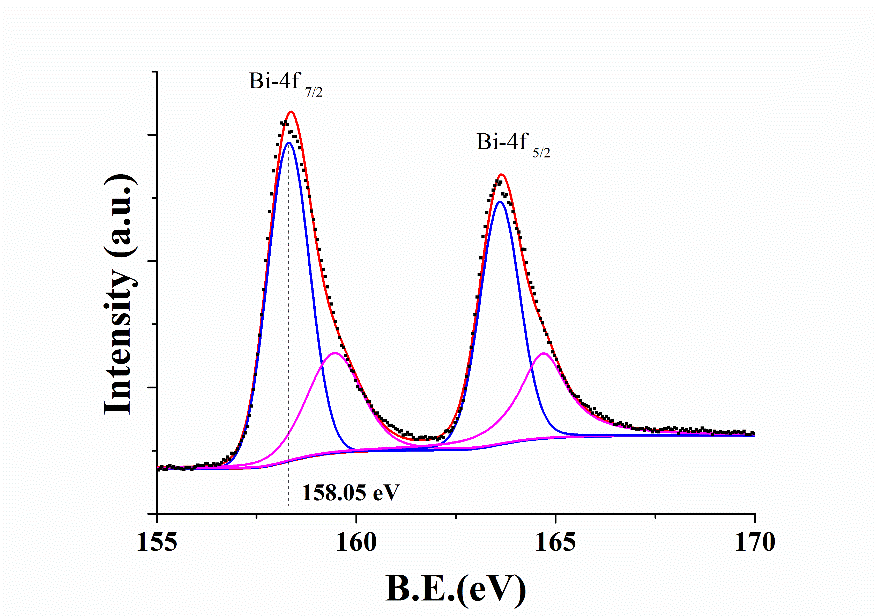


**Figure S5.** XPS of Bi-4f core level for Bi-Ir pyrochlore.


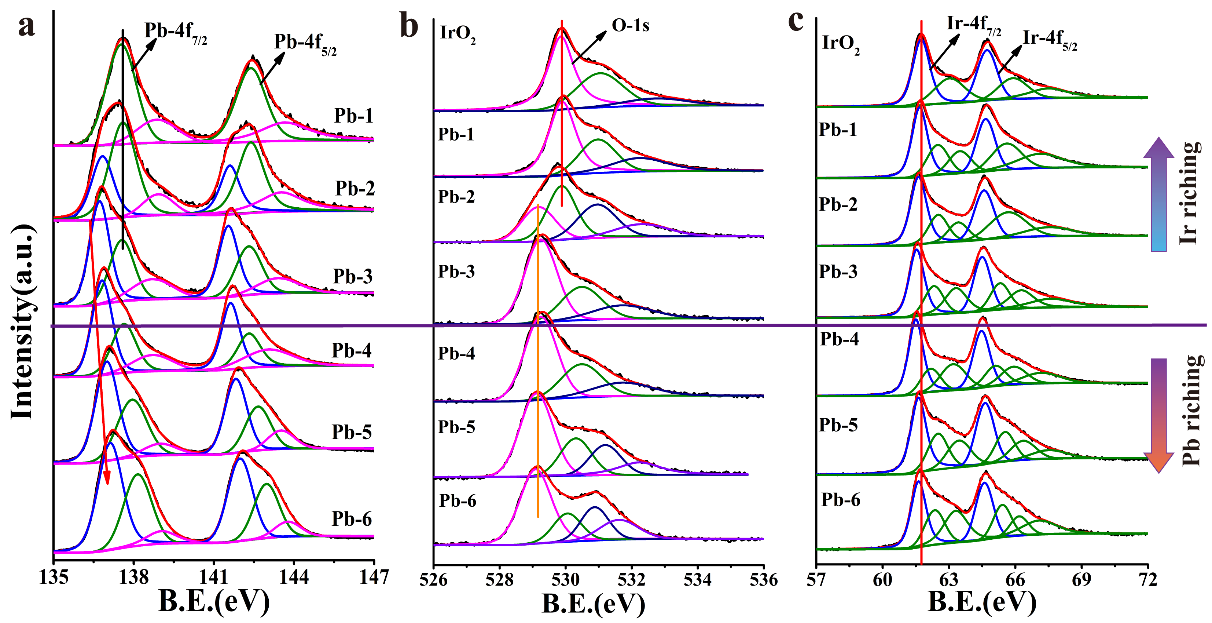


**Figure S6.** XPS spectra of Pb-4f, O-1s and Ir-4f core levels in different Pb-Ir oxides, Pb-4 corresponding to Pb-Ir pyrochlore. From Pb-4f XPS, it is clearly noted that two de-convoluted peaks were identified in Pb-Ir oxides except Pb-1. These doublet peaks are also observed in β-PbO_2_, but their peak shapes are different. For later it is a tetragonal structure, while in here, Pb is located in cubic pyrochlore structure. Their different crystal structure is responsible for their novel peaks distribution. The O-1s are also complicated, in Pb-2 case, three fitting peaks are observed, the low energy one may contribute to Pb-Ir pyrochlore, while the middle one arise from binding of Ir atom which is consistent with IrO_2_ reflection. To be noted, there is no significant difference in energy position of lattice O between pyrochlore and fluorite structure. For the Ir-4f XPS, there is a slight shift to lower energy in all Pb-Ir oxides even in Pb-Ir fluorite.


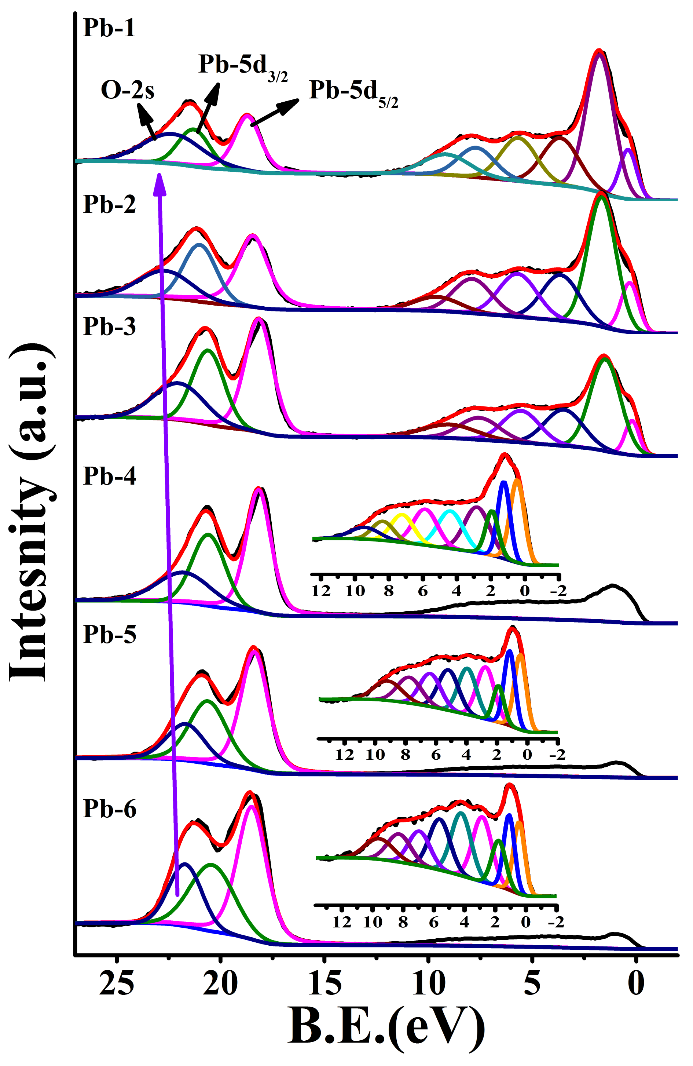


**Figure S7.** VBXPS for all prepared Pb-Ir oxides. Noted that in valence bands, the d bands intensity (0-10 eV wide) mainly from Ir-5d progressively decreases with reducing Ir contents. It implies that Pb-6p has little contribution to the valence band intensity. The energy of O-2s peak is decreasing with more Pb contents, which is consistent with O-1s change. For the Ir-5d valence band, it can be fit by a shoulder peak near the Fermi level which is regarded as J=1/2 subband from t_2g_ orbitals splitting, however, this shoulder peak disappear in Pb-4, Pb-5 and Pb-6 cases, because they are pyrochlore and fluorite structures not rutile as IrO_2_. This shoulder peak can be observed in Pb-2 and Pb-3 cases as they comprise some IrO_2_ oxides.


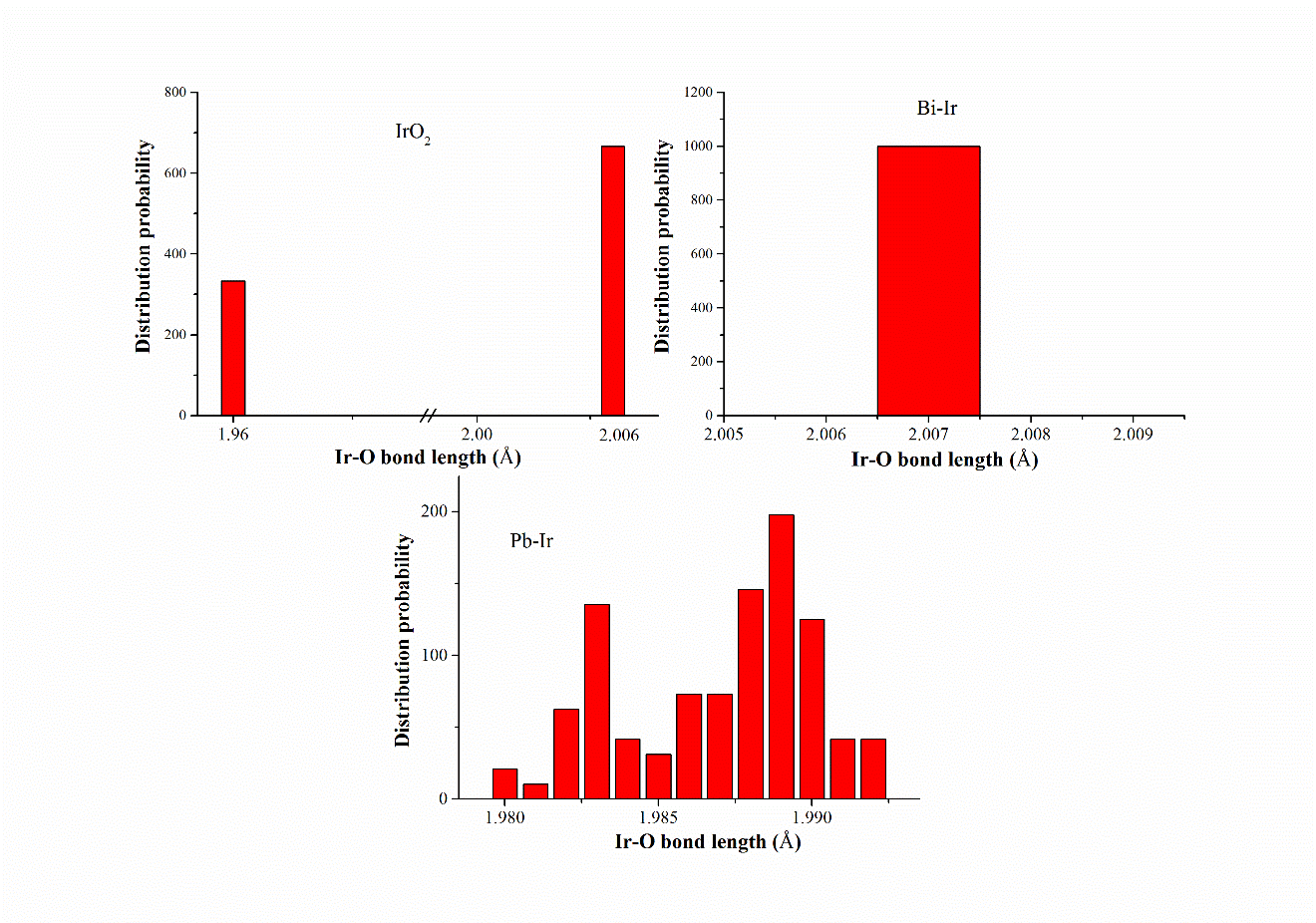


**Figure S8.** Distribution probability of Ir-O bond lengths in IrO_6_ coordination by optimizing the unit cells of IrO_2_, Bi-Ir and Pb-Ir pyrochlores. It can be concluded that IrO_6_ geometry in IrO_2_ is a compressed one with D_2h_ symmetry, whereas for Bi-Ir pyrochlore is a regular one with O_h_ symmetry. However, for Pb-Ir, it is a distorted one due to Ir-O bonds having various possibilities.
